# Understanding complexity in neurodegenerative diseases: *in silico* reconstruction of emergence

**DOI:** 10.3389/fphys.2012.00291

**Published:** 2012-07-23

**Authors:** Alexey Kolodkin, Evangelos Simeonidis, Rudi Balling, Hans V. Westerhoff

**Affiliations:** ^1^Luxembourg Centre for Systems Biomedicine, University of LuxembourgEsch-sur-Alzette, Luxembourg; ^2^Institute for Systems Biology, SeattleWA, USA; ^3^Department of Molecular Cell Physiology, VU UniversityAmsterdam, Netherlands; ^4^Manchester Centre for Integrative Systems Biology, FALW, NISB, The University of ManchesterUK; ^5^Synthetic Systems Biology, SILS, NISB, University of AmsterdamNetherlands

**Keywords:** systems biology, systems biology diseases, network diseases, weak emergence, strong emergence, computer modeling, neurodegenerative disease, Parkinson's disease (PD)

## Abstract

Healthy functioning is an emergent property of the network of interacting biomolecules that comprise an organism. It follows that disease (a network shift that causes malfunction) is also an emergent property, emerging from a perturbation of the network. On the one hand, the biomolecular network of every individual is unique and this is evident when similar disease-producing agents cause different individual pathologies. Consequently, a personalized model and approach for every patient may be required for therapies to become effective across mankind. On the other hand, diverse combinations of internal and external perturbation factors may cause a similar shift in network functioning. We offer this as an explanation for the multi-factorial nature of most diseases: they are “systems biology diseases,” or “network diseases.” Here we use neurodegenerative diseases, like Parkinson's disease (PD), as an example to show that due to the inherent complexity of these networks, it is difficult to understand multi-factorial diseases with simply our “naked brain.” When describing interactions between biomolecules through mathematical equations and integrating those equations into a mathematical model, we try to reconstruct the emergent properties of the system *in silico*. The reconstruction of emergence from interactions between huge numbers of macromolecules is one of the aims of systems biology. Systems biology approaches enable us to break through the limitation of the human brain to perceive the extraordinarily large number of interactions, but this also means that we delegate the understanding of reality to the computer. We no longer recognize all those essences in the system's design crucial for important physiological behavior (the so-called “design principles” of the system). In this paper we review evidence that by using more abstract approaches and by experimenting *in silico*, one may still be able to discover and understand the design principles that govern behavioral emergence.

## Introduction

### System design in habitual and systems biological modelling

In spite of great effort, the pathophysiology of many diseases remains unclear. In this paper, we focus on complicated, network diseases, such as neurodegenerative diseases, in an effort to identify the obstacles to understanding the mechanisms and processes behind their development. Diverse phenomena appear to be involved, the connections between them being unclear.

Our effort towards rationally understanding reality usually translates into the need to model this reality. Here we use “modeling” in the broad sense, where a “model” is defined as a “representation of a limited part of reality by related elements,” a projection of a system, e.g., the real world, into another system; the construction of physical, conceptual or mathematical simulations of the real world. We may say that the way in which we see the real world is just a model of it; it is our interpretation of this world based on our theories. In fact, we do this type of modeling in everyday life. Moreover, a lot of our everyday modeling is about the function of some system, or, to be more precise, about the reality that we perceive as a system. Then, in our minds, we make a connection between the interacting components of the system and its emergent properties and, even solely by intuition, we may end up with an explanation of how the organization of the system ensures its functionality: an explanation of the design of the system.

Design can be defined as “the constellation of system components, their specific properties and their pattern of interactions that together determine the integrated behavior of the system” (Wall et al., 2004). In biology, this integrated behavior corresponds to function and fitness. This understanding of how the way of arranging the system is responsible for a generic set of functions, may be summarized in the more general concept of a design principle. An explanation of the design of a system builds a bridge between the underlying mechanism of interacting components and a certain emergent property that is associated with the design. In this respect, the design principle resembles the causal explanation, but it also adds a new perspective by requiring the emergent property to be functional, i.e., to play a certain role; it also relates to the teleological explanation.

Let us consider a bicycle as an example. When we say “bicycle,” in our minds we see a system consisting of several components (wheels, brakes, steering bar, etc.). Rather intuitively, we build a bridge between the interacting components and the emergent property—the functioning of the system as a whole: the bicycle moving—and use this model to understand a possible dysfunction of the system when some interactions between system components do not work properly or when some components are completely removed. For example, we can easily imagine what will happen if the bicycle chain breaks.

In biology, we proceed with similar mental processes and this works relatively well when we have a clear picture of the system in mind. This is the case for some genetic diseases. We can easily make the following associations: defect in gene → defect in protein → variance in emergent property → defect in function. But, as in the example of neurodegenerative diseases, dysfunction may well be more complicated; we may not be able to narrow down the cause of the disease to one defective protein. Instead, we may have to view the disease as a shift of homeostasis and to accept the idea that homeostasis is shifted away from its normal range, due to a large set of perturbations in the network of interacting biomolecules in the entire organism (Del Sol et al., 2010).

### Parkinson's disease (PD) as an example

Protein aggregates, such as senile plaques and neurofibrillary tangles (regarded as extracellular deposits of amyloid in the gray matter) and complexes of hyperphosphorylated tau protein, relate to many neurodegenerative diseases (Tiraboschi et al., 2004; Lee et al., 2011), as do inflammation, degradation of the interactions of neurons with glial cells, and mitochondrial dysfunction. Here, we focus on the latter and on excessive ROS generation, which plays a role in the death of dopaminergic neurons in Parkinson's disease (PD) (Choi et al., 2011; Mccoy and Cookson, 2011; Selivanov et al., 2011). Mitochondria are in a continuous growth and apoptosis cycle. Normally, electrons pass through the electron transfer chain (ETC) in mitochondria, from complex I to complex IV, where they reduce molecular oxygen (O_2_) to water. Some electrons escape from the ETC before reaching complex IV. These escaped electrons reduce oxygen to other ROS, which are continuously neutralized by super oxide dismutase (SOD) and consumed by various antioxidants. Still, some ROS can escape and cause an oxidative chain reaction resulting in mitochondrial damage. The latter activates even more ROS generation and finally leads to mitochondrial apoptosis (Brady et al., 2004).

**Figure 1:**
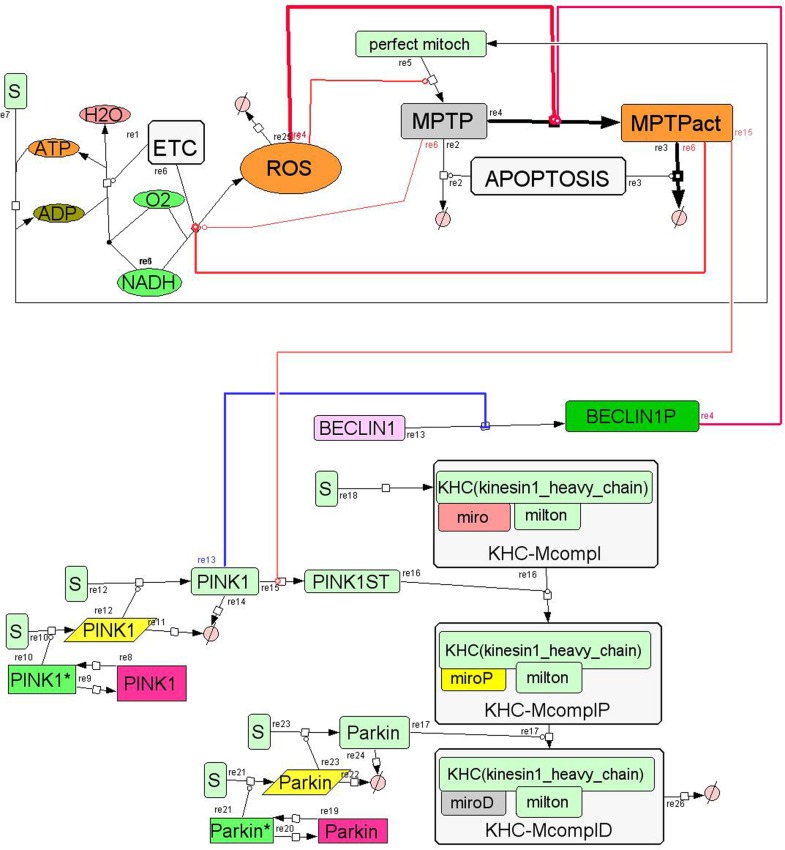
**Proposed core mechanism for modeling of growth and apoptosis of mitochondria.** The basic assumption is that mitochondria can be in one of three different states: normal mitochondria, damaged mitochondria with non-active mitochondrial permeability transition pore (MPTP), and damaged mitochondria with active MPTP (called MPTPact on the diagram). Activation of the MPTP increases apoptosis of mitochondria (Brady et al., 2004). ROS is produced by the mitochondria and is consumed, e.g., by antioxidants. ROS activates mitochondrial damage and the MPTP. ATP is produced by the functional mitochondria only (where MPTP is not activated). ATP is used for the synthesis of mitochondria. BECLIN1 activates MPTP and PINK 1 inhibits BECLIN1 (Cui et al., 2011). The core mechanism is open to incorporating other components, e.g., Miro and Parkin.

If the cell was absolutely passive, than this would be the end of the story. But the cell has a kind of “intelligence”; it is able to sense the increase of ROS concentration and counteract this pathological condition e.g. by activation of the synthesis of antioxidants or removal of damaged mitochondria. The latter involves complicated regulatory network mechanisms with many feed-back and feed-forward loops. Figure 1 presents a simplified scheme of a small part of this regulatory network. In reality, many more components and feed-back loops comprise the system. For example, BECLIN1 activates the membrane permeability transition pore (MPTP) and PINK 1, which in turn inhibits BECLIN1 (Cui et al., 2011). There is also a mechanism of apoptosis of mitochondria which involves Keep1, Nrf2, Bach and other components. MPTP stabilizes PINK1 in the mitochondrial outer membrane and PINK1 phosphorylates Miro; later Parkin degrades phosphorylated Miro, which arrests the motility of damaged mitochondria (Wang et al., 2011).

The mitochondrial component of the disease should be integrated with processes such as protein aggregates and inflammation. For a more complete picture, these processes could additionally be integrated with the pathological process in the micro flora of the gut, which contribute to the development of neurodegenerative diseases (Forsyth et al., 2011; Renz et al., 2011). As is evident by even this short summary, it is easy to get lost in the maze of processes and interactions taking place in the pathology of a neurodegenerative disease. Which one of these observed processes is the cause and which is the consequence? Why does one person get the disease and another person does not? Which gene mutations contribute to the disease? To date, we have still barely scratched the surface in our effort towards understanding the mechanisms of complex diseases. The problem may even go deeper than this: can we even be certain what we mean when we talk about “understanding”? And, is the understanding we would talk about operational, achievable and useful?

### Understanding disease using the network approach

The perception of a disease as a network perturbation (Hornberg et al., 2006) has two important implications: first, there may be multiple perturbations that could result in a single disease phenotype; and this agrees with our understanding of many complex diseases. Second, the converse argument predicts that there could be multiple ways to return the network back to the “healthy” attractor; and targeting several points in the network may be the most efficient way in which to achieve this homeostatic shift. This provides the conceptual basis for the action of polypharmacy, or multi-drug therapies (MDT) (Luni et al., 2010; Volpe et al., 2010) and the development of network targeting drugs (Bakker et al., 2002; Borisy et al., 2003; Lehar et al., 2009).

In the network approach we have to deal with very complex network structures that may be too complicated to comprehend in a “naked,” i.e., unaided brain. It is thereby logical that we always try to simplify our model to something rationally understandable. This is what biology has been trying to do for centuries, sometimes rather successfully. There are at least three serious reasons why such an approach may fail, however. First, as we have seen in the example of neurodegenerative diseases, the simplification might not work if a disease is caused by too many factors, both genetic and environmental; in essence, the simplified model is too far from reality. Second, the simplification might not work because, e.g., the substrate of a single enzyme is not necessarily the metabolite causing the disease, but may belong to a pathway that is impacted by the disease-causing mutation; we thus need to determine cause versus consequence associations. Third, the reduction of the model might prevent the identification of the important underlying design principles of the network, meaning that we might miss the potentially most powerful therapeutic targets if aiming to restore the network to normal homeostasis.

One solution is to give up on our ambition to simplify complexity and accept the limitations of our brain. Three centuries ago, Antony van Leeuwenhoek accepted the limitations of human eyes and enhanced our vision with the invention of the microscope. Today, we need to accept the limitation of the human brain and delegate the modeling (and also the understanding) of reality to the computer and even more sophisticated Information and Communication Technologies (ICT) (Lehrach et al., 2011). Indeed, interactions between biomolecules are mostly physical and chemical reactions; they are measurable processes and it should therefore be possible to describe them through mathematical equations. These equations can be integrated into a mathematical model and simulated on a computer (Snoep et al., 2006; Westerhoff et al., 2009a). Such an approach can break through the limitations of any single human mind to perceive the extraordinarily large number of interactions and parameters that are involved, and in fact serve the nonlinear integration of the activities of a large number of human brains. The science aiming to meet these expectations was born about 10 years ago and is named bottom up systems biology (Westerhoff et al., 2009a).

We would like to emphasize that systems biology is not just computation plus experimentation, and it is not just mathematics plus biology either. Systems biology is a conceptual approach for understanding biological complexity as such, in terms of interactions between huge numbers of macromolecules (a number much bigger than what a human brain can perceive). Mathematical descriptions and computer simulations are tools that may be used in this approach. Systems biology is a new science with new paradigms (Westerhoff et al., 2009a) not identical to any of, but arising from the integration of, physics, chemistry, and the life sciences, with the help of mathematics, in order to reconstruct and understand biological emergence *in silico*.

The success of systems biology would literally mean that we do not have to hold a whole model of the processes we study in our brain. Instead, we would have a computer replica of the organism; we would reconstruct the biological emergence *in silico* (Kolodkin et al., 2011a). With respect to a human organism, this approach would lead to a mechanism-based computer replica of the whole body – the so-called silicon human. In order to consolidate efforts in that direction, the Tokyo declaration (Hunter et al., 2008) was signed by leading systems biologists in February 2008, with the aim for a computer replica of a whole human body to be 90% complete by 2038. The whole-body model approach does not come without obstacles that need to be overcome. The main concerns usually refer to the “astronomical” number of interactions involved in the complete body (Noble, 2006). However, because of the modular organization of the organism, the number of interactions may be large, but not quite “astronomical.” Let us show what difference modularity makes for the numbers. If we talk about a human being, and think about the interactions between the 25,000 genes in each of the 10^14^ cells of the whole body (2.5×10^18^ genes per body), then the number is pretty high, i.e., 2.5 × 10^18^!/2 ≈ 10^2×10^19^, i.e., a 1 with 2 × 10^19^ zeros, much more than the number of atoms in the universe (≈ 10^80^). If we only envisage binary interactions, the number is smaller (2.5 × 10^18^ × (2.5 × 10^18^−1)/2 ≈ 3×10^36^) but still enormous. However, taking into account the modular organization of the body and the fact that not everything may interact with everything else, the number of interactions becomes much smaller. Let us start with a single cell. If a cell contains about 1,000 metabolic enzymes (‘enzyme types’ really but we assume that all enzymes defined by the same gene(s) behave as a single ensemble) and about 500 metabolites (‘metabolite types’ really but we again assume ensemble behavior), maximally 5 × 10^5^ binary enzyme-metabolite interactions are possible. These are the current numbers for yeast (Herrgård et al., 2008), but although the yeast genome is approximately five times smaller than, e.g. the human genome, we do not expect a high difference between organisms in terms of the number of catalyzed reactions in a single cell. Besides, 5 × 10^5^ interactions are an overestimation since in reality not every enzyme can interact with every metabolite. It is much more likely that an enzyme interacts on average with at most five metabolites, bringing down the number of metabolic interactions to only 5000. Continuing this line of thought, there are about 3000 human transcription factor genes. If every transcription factor binds to 100 different genes, then there are about 3,000 × 100 = 3 × 10^5^ interactions. If the average factor is much more specific, then this number could be only 10,000. Together with metabolic interactions, we approach the order of 10^4^. The addition of tens of thousands of interactions on the level of transporters, receptors and so on would not change this order of magnitude of the number of interactions in a cell substantially. Now let us go to the intercellular level where 10^14^ cells are organized in tissues and organs, five cell types per organ. Let us say that each cell type interacts with 100 neighbors via maximally 50 metabolites (25,000 interactions), and that one organ interacts with all other 71 organs via another 50 metabolites (a little more than 3500 interactions). If we sum all interactions mentioned above, we would be still in the order of 10^5^. This is indeed not a small number, but taking into account the increasing computational power, we do not see that it should cause any principal limitation. The essence of these calculations is that, if one foregoes the natural organization of living systems, the number of interactions appears astronomical, but with a bit of realism, these numbers turn out to become manageable. Another concern is the lack of experimental information, e.g., concerning the kinetic parameters for these interactions. Nevertheless, as discussed already, the number of interactions in the human body is finite and, taking into account the huge leaps of progress in experimental biology, we might anticipate that, in the future, much of the information concerning these interactions will eventually become available for the building of the silicon human.

As we have mentioned above, in everyday mental modeling we bridge the network of interacting components and the emergent properties of the system intuitively. On the contrary, in systems biological modeling, the emergence is reconstructed entirely in a computer model. Consequently, the intuitive grasp might be lost. Relating the components of the network to the emergent properties might require more abstract understanding. Perhaps we should first define more clearly what emergence is, classify different types of emergence, and analyze the limitations of the reconstruction of emergence.

## Discussion: towards reconstruction of emergence and understanding of design

### Weak and strong emergence

In a metaphysical setting, an emergent property of a system is defined as a property which satisfies three criteria, which we will summarize as three theses or notions: (i) the thesis of being a systemic, organizational property (a property that should not be exhibited by elements in isolation); (ii) the thesis of physical monism, which restricts the nature of the system's elements, states the certification that the system consist of only physical entities and denies any supernatural influences and (iii) the thesis of synchronous determinism, which restricts the way of how systemic properties and the system's microstructure are related to each other and states, i.e., there can be no changes in systemic properties without changes in the structure of the system or in the properties of its components (Stephan, 2006). This definition of emergence is of course open to debate. For instance, according to the above definition, almost all properties of any system could be considered emergent. Intuitively, we note the difference between the emergence of self-consciousness in human behavior and the emergence of hardness in diamonds. The philosophy of mind suggests making a distinction between *strong emergence* (self-consciousness) and *weak emergence* (hardness of a diamond) (Stephan, 2006) by the criterion of *irreducibility*. In the words of the philosopher Broad, irreducibility means that “… the characteristic behavior of the whole could not, even in theory, be deduced from the most complete knowledge of the behavior of its components, taken separately or in other combinations, and of their proportions and arrangements in this whole (Broad, 1925)”. According to the contemporary philosopher Stephan, a systemic property is irreducible if it satisfies at least one of the following criteria: “ if (i) it is not functionally construable or reconstruable, if (ii) it cannot be shown that the interactions between the system's parts fill the systemic property's specified functional role, or if (iii) the specific behavior of the system's components, over which the systemic property supervenes, does not follow from the components behavior in isolation or in simpler configurations” (Stephan, 2006).

The emergence of a network disease such as PD would follow the classification of strong emergence exactly because of the third criterion of irreducibility. The disease might be functionally construable or reconstruable, i.e. it can be shown that the interactions between the system's parts (genes, proteins, etc.) fulfill the systemic property's specified functional role, but, the specific (diseased) behavior of the system's components (biochemical network of the complete organism), over which the systemic property supervenes, does not “follow” from the components behavior in isolation (properties of proteins in isolation) or in simpler configurations (isolated part of the network of interacting biomolecules). Of course, it is important here to define what “follow” means. If it were to refer to reconstruction, then the third criterion would become tautologous with the first. For the philosophy of systems biology (Boogerd et al., 2007) it may be useful to define “follow” as “follow with the naked brain.” If a computer replica is needed to reconstruct the emergence by replaying the behavior and interactions of all the components, then this will go far beyond our brain being able to *follow* emergence. If a systemic property is strongly emergent, this does not imply that this property cannot be reconstructed in a mechanistic model, it merely means that our brain cannot understand the emergence.

The reason why our brain cannot understand emergence is that properties of *in vivo* components cannot be seen separately from the system. For instance, the behavior of a certain protein in the body depends not only on its own properties, but also on whether other proteins such as activators, inhibitors or chaperons are present, how all other proteins in the system change, pH, macromolecule crowding, etc. *In vitro*, binding affinity of a substrate for its isolated enzyme is a property of that enzyme. *In vivo* however, it depends on all other enzymes in the system because the latter determine the pH and the affinity depends on pH. Properties of the protein of interest depend on the state of the system as a whole; in biological systems, component properties are state-dependent and therefore system dependent (Boogerd et al., 2005).

A reason to interpret the property of strong emergence in this way is to rid the discussion of the issue of vitalism or intelligent design. Without the limitation of the definition of “to follow” to what we propose here, strong emergence would correspond to the admission of vitalism or “intelligent design” to systems biology and biological design. Vitalism and intelligent design should be kept out of these discussions of systems biology and modern biology, as they make these disciplines nonoperational.

Different criteria may contribute to state-dependency and each of these criteria may provide a possible measure for evaluating the strength of emergence. The first criterion may be the number of interactions leading to the emergence. For example, the proliferation of a healthy cell could be considered more strongly emergent than the proliferation of a tumor cell, if proliferation of normal cells were determined by more regulatory processes than that of tumor cells.

The second criterion is thermodynamics, which is connected with the flux of energy through a cell. When a living organism grows or even when it maintains itself, it requires free energy dissipation to make its processes proceed at sufficiently high rates (Glansdorff et al., 1974; Westerhoff and Van Dam, 1987). Consequently it requires a high flux through a catabolic pathway such as the pathway converting glucose to pyruvate. We cannot reconstruct therefore the ability of a cell to exist without at least qualitative information regarding this flux (Boogerd et al., 2007). The knowledge of steady-state concentrations of intermediates is not enough, because steady-state concentrations could be the same for different values of the flux.

A third criterion may be connected with the occurrence of hysteresis in a system, which restricts possibilities of predicting the system's state without looking at its history, largely because it is difficult to know all relevant details of its present state. Even though biological systems should be Markovian in principle, insufficient detail may be known for one to understand them in exclusively Markovian terms. A second realm where hysteresis may be important is that of bi-stability and irreversible transitions.

For the time being, the above criteria for the evaluation of the strength of emergence are just suggestions for further thinking. There is still no solid theory on how to estimate quantitatively to what extent “strong” emergence is strong. It is not our current goal to arrive at an over-arching theory of how to integrate the above, and most probably other criteria into a measure for the overall strength of emergence. We discuss this with a different purpose: to show that there may be a gradation of how strong emergence is (Kolodkin et al., 2011b).

### Ways to reconstruct emergence, including strong emergence

There are various approaches towards the mathematical description of interactions in biological systems. The simplest one may be the one based on graph theory. A graph is a set of objects called nodes or vertices connected by links called lines or edges. Nodes might be attributed to different species of biomolecules and edges to interactions between these biomolecules. If a line from node A to node B is considered to be identical to a line from B to A, a graph is called an undirected graph. If the two directions are counted as being distinct arcs, a graph is called a directed graph (Kestler et al., 2008).

In quantitative mechanistic models, one needs to describe interactions between species quantitatively and accurately, e.g., in terms of mass action or Michaelis-Menten kinetics. Then the model describes the kinetics of the system, e.g., changes in the concentrations of the variable intermediates as functions of time. If reaction rates in a kinetic model are based on the real ensemble averaged (thermodynamic) properties, this kinetic model could also be called a (non-equilibrium) thermodynamic model (Bruggeman and Westerhoff, 2006).

Models can be either global (referring to a network of subsystems such as organs) or detailed (referring to a network of molecules). If detailed on the level of molecules, we further distinguish between macro-, meso-, and microscopic modeling of ensemble averages. For example, if we neglect the limitations imposed on the reaction rates by the required diffusion of molecules and consider each species of biomolecules as a single pool, then the model would be called macroscopic and could be described as a system of ordinary differential equations in terms of thermodynamic variables (Westerhoff and Van Dam, 1987). Mesoscopic models require stochastic simulations using the master equation and formulations in terms of probability functions (Bruggeman et al., 2009). Microscopic models trace every molecule individually (Andrews et al., 2010).

The final goal of systems biological modeling is to link the layer of interacting biomolecules with the strong emergence of systemic functioning of the organism. There are three different strategies to build this link. One way is the bottom-up, mechanism-based strategy: first, one describes the actual mechanism in terms of mathematical equations, then one assigns model parameters with experimentally determined values and lastly one verifies the model by comparing its systemic behavior with the behavior of a real system (Bakker et al., 1997, 2000; Rohwer et al., 2000; Westerhoff, 2001). The term “bottom-up” refers to the direction chosen: from known or assumed properties of the components one deduces system functions (Westerhoff, 2001; Reijenga et al., 2005; Kartal et al., 2011).

Another way is to start with the systemic behavior (top-down modeling): first, one determines how the (often complicated) systemic function of interest varies with conditions, or with time, and from these observations one induces hypothetical structures that can be responsible for this function. This is a data-driven, “digital” approach (Lauffenburger, 2000). System behavior is influenced (perturbed) and a top-down, bird's eye view is taken, looking “down” towards system components, on a genome-wide, proteome-wide or metabolome-wide scale. There is yet another top-down approach, which starts from physiology rather than genomics and from macroscopic, often physical properties, such as force and length, pressure and volume (Hunter et al., 2008). This has been called the Virtual Physiological Human approach (VPH).

A complex, small system, say a metabolic network consisting of biomolecules and exhibiting its own emergent properties, can be considered at the same time as a part of a larger system, like the cell. In turn, cells interact with each other and form an even more complex system, for example an organ, and so on. Consequently, the fragmentary knowledge can also be integrated in a third, so-called middle-out strategy that allows modeling the behavior of a single organ or a single functional system in terms of interactions between entities which do not necessarily belong to the same molecular level of organization. These entities could be biochemical pathways, or organelles, or cells, or even entire organs (Noble, 2006). The above approaches already overlap to significant extents, e.g., the VPH approach shares the heart as major focus with the middle-out approach. And in the recent ITFoM initiative (Lehrach et al., 2011) bottom-up molecular systems biology and the VPH approach integrate.

Availability of data concerning single interactions and knowledge of hypothetical mechanisms drive the bottom-up strategy while the development of bioinformatics and the availability of large sets of measured variables drive the top-down strategy. At least one goal of the three approaches is the same: to link the underlying layer of interacting molecules with the physiological behavior. For instance, when the systemic function in the middle-out approach is extended to the whole organism and when the underlying level of interacting component reaches the level of physico-chemical interactions between biomolecules, the model should be equivalent to one obtained by use of the bottom up or top-down strategy. Analogously, the perfect top-down parameterization would create a model with the same functionality as a model built using the bottom-up approach. If taken to their (perhaps unattainable) extreme, it should not matter which approach is used; the final aim is a unique computer replica of the living organism. However, models built with top-down approaches are phenomenological; consequently every new experiment would require refitting the entire model. A model based on the bottom-up approach, such as the silicon cell model, is free from this drawback. Once known, the mechanism of interactions between components and parameter values should not change anymore, unless the model is wrong. If a module is modeled correctly, it could always be incorporated as an “object” into a bigger model. Building of the final, large-scale model would merely mean the adequate interconnection of many existing “objects.” One simple model could then be integrated with other simple models and should be amenable to stepwise expansion via inclusion of additional network components. Allegorically, the first object becomes a “crystallization point” with the potential for further “growth.” This might seem to make the bottom-up approach superior. However, this approach has the disadvantage that, for large numbers of pathways and networks, the required mechanistic component knowledge is unknown and hard to obtain, at least for the foreseeable future. Where implemented, the approach has not yet led to complete agreement between systems level prediction and experimental result (Teusink et al., 2000).

### A new look at design as another side of state dependency of emergence

If we wish to understand the mechanism of strong emergence as it is, or if we want to reconstruct strong emergence without deeming it to be less strong, we need to know about all interactions and we need information concerning all state-dependent properties of system components. Alternatively, perhaps we can identify more general rules for the arrangement of system components in the whole vis-à-vis its functions—the so-called design principles (Wall et al., 2004). A design principle is a kind of knowledge about the state dependency of the component properties. Then, concepts of emergence and design meet each other. We can rephrase this by saying that the reconstruction of a strongly emergent property requires both (i) the knowledge of the system's components in isolation and (ii) the design principles used in the building of this particular system.

A design study is not necessarily associated with computer modeling. Design principles can be discovered in thought experiments as well. For example, one may ask why tetrapods have lungs and find several hypothetical advantages of having lungs. These speculations may bring us to explanations for a design which could be generalized to design principles (Wouters, 2007). However, thought experiments are rather speculative and lack objective criteria justifying that the design of an organism without a certain trait, e.g., the design of *Acanthostega* (an extinct tetrapod with gills instead of lungs) is less advantageous. It is difficult to bring *Acanthostega* back to life in order to answer such questions through experimentation. A solution is to build a model of this organism and to reconstruct its emergent properties *in silico*. This is quite possible. The advantage of confronting different designs *in silico* is that it affords us the possibility of using strict, mathematically controlled comparison. According to wall, Hlavacek and Savageau (Wall et al., 2004), mathematically controlled comparison is based on considering each design as a special case of the same model. Then, each design will correspond to a region in the parameter space and *a priori* mathematically precise conditions may be applied when comparing systems selected from these regions, so that “the systems under comparison can differ in ways that are essential for distinguishing system types but are otherwise constrained to be as similar as possible”. For example, systems are allowed to have differences in transcriptional control so that systems of different forms of coupling can be compared, but are constrained to be identical with respect to translation. If differences in functional effectiveness are observed, the various system types are associated with optimal solutions for distinct regulatory problems. If no differences are observed, variations among system types are considered to be neutral.” (Wall et al., 2004). These ideas can be used to develop a procedure for discovering design principles of the system reconstructed *in silico* (Kolodkin et al., 2010) and have been applied for identifying design principles in various systems. Next, we discuss one such example.

### An example of *in silico* reconstruction of emergence and of the discovery of system design principles

We discuss an example of how emergent properties of the system may be reconstructed in a kinetic model and how this reconstruction of emergence can be used for understanding design principles of the system. For this, we turn to the field of nuclear receptor (NR) signaling. However, we anticipate that similar approaches will prove useful for other systems, including those determining disease.

**Figure 2:**
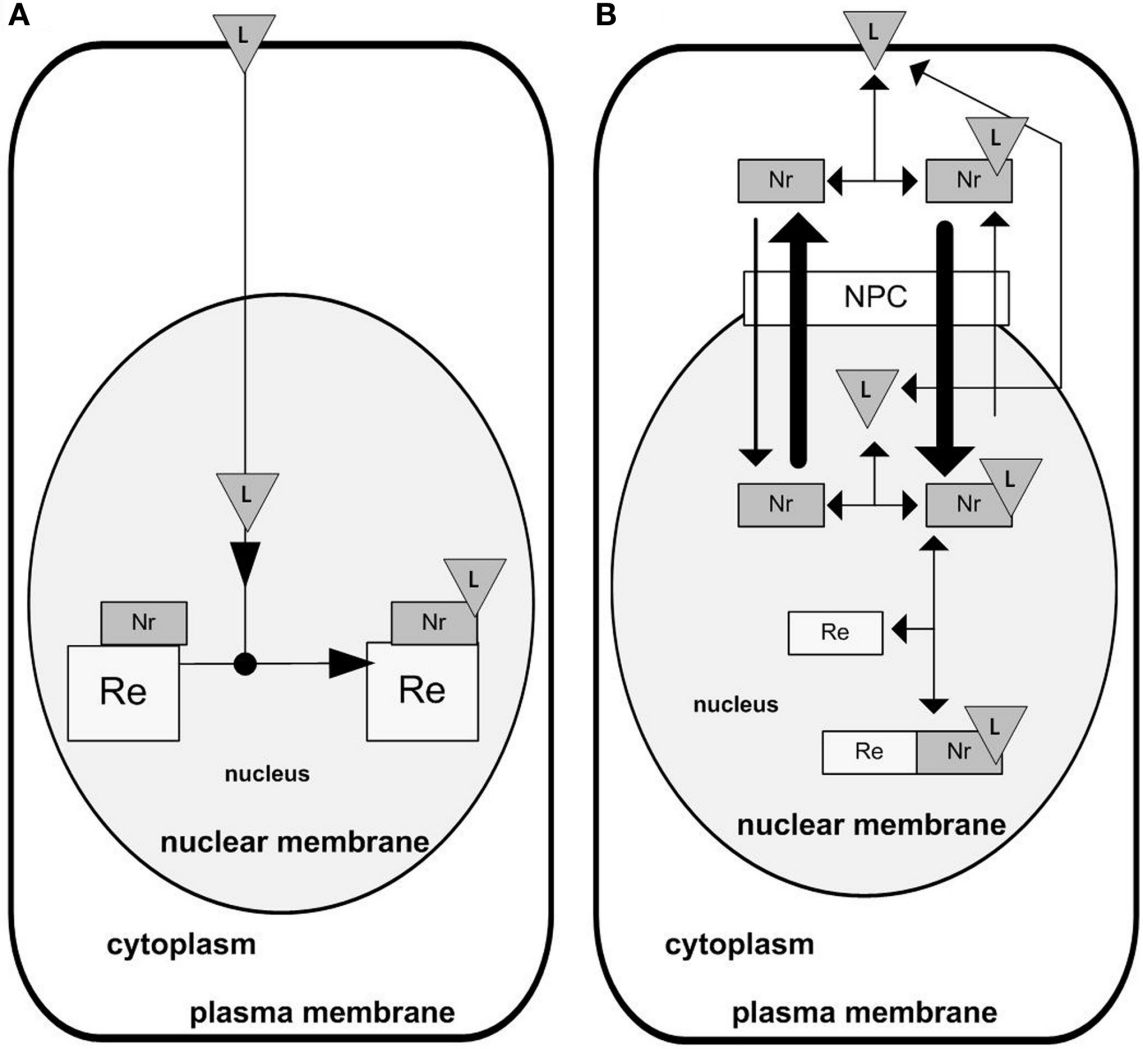
**Designs of NR signaling. (A)** Classical design. The NR is attached to its response element on the DNA and waits for a ligand freely diffusing to the nucleus. Upon ligand binding the NR participates in chromatin remodeling regulating the availability of genes for transcription initiation. (**B)** Design inferred from the detailed NR signaling network. There is an active nuclear import and export of the NR, with core-NR having lower import into the nucleus than the receptor bound with its ligand (NRL).

NRs are involved in a diverse range of regulatory functions, such as in development, cellular growth, inflammation and metabolism (El-Sankary et al., 2001, 2002; Phillips et al., 2003; Aouabdi et al., 2006; Carlberg and Dunlop, 2006; Ebert et al., 2006; Cutress et al., 2008). NRs work as transcription factors, the activity of which is regulated by intra- and extracellular signaling molecules. Many of these molecules are lipophilic compounds such as steroid hormones (ligands for glucocorticoid receptors) and vitamin D (Carlberg and Dunlop, 2006; Ebert et al., 2006; Cutress et al., 2008). Hydrophobic, extracellular signal molecules serving as NR ligands are able to diffuse through the plasma membrane, the cytosol and gain entry to the nucleus (Gardner, 1975). There they are able to bind to the corresponding NRs, which are already bound to their specific DNA binding site called a response element (RE) (Figure 2A). This mechanism may be grasped in the design shown on Figure 2A. However, we may also consider more components involved in the NR signaling, for example importin and exportin proteins which allow shuttling of the receptor between the nucleus and cytoplasm. Taking into account the nucleo-cytoplasmic shuttling of the receptor, the network might be redrawn as shown on Figure 2B (Kolodkin et al., 2010).

In this design (Figure 2B), active nuclear import of the core-NR is lower than the nuclear import of the receptor bound with its ligand (NRL). On the contrary, the nuclear export of liganded receptor (NRL) is higher comparing to the core receptor.

If both network designs (Figures 2A,B) are transformed into mathematical kinetic models, at least some of the emergent properties of the two systems would be different, but certain emergent properties of the real life system are not displayed by the network design in Figure 2A, e.g., the translocation of the receptor into the nucleus upon the addition of ligand. Consequently, we consider the more complex design to be more realistic. Then, the question can be raised: why is the real system organized in the way it is, i.e., according to the more complex design shown on Figure 2B and not differently, e.g., not simpler? The model allows us to explore this by simulating the emergent behavior of the network with hypothetical designs and to determine which design is the most advantageous. With respect to the example presented here, simulation has shown that the design in Figure 2B is the most advantageous, as only this design provides active pumping of the ligand into the nucleus (Kolodkin et al., 2010). It was hypothesized that the latter becomes possible because the NR does not work as receptor only, but also as a smart “ferry-boat” transporting the ligand into the nucleus. When the receptor is liganded, it is transported mostly into the nucleus. When the receptor is free, it is transported mostly out of the nucleus. Consequently, the ligand is actively pumped and might be accumulated in the nucleus, which would allow a higher concentration of the liganded receptor bound with the RE on the DNA (NRL), a higher and quicker transcriptional response, and, as a result, may help increase responsiveness and sensitivity of NR signaling (Kolodkin et al., 2010). The reconstruction of the emergent properties of the network allowed us not only to identify the best design, but also to explain its advantages and, by these means, to understand the mechanisms underlying the functioning of the system.

### The complexity of model reconstruction and the strength of emergence

Since the same biological object might be described by various models, e.g., with a higher or lower number of interactions, this implies that the same property of an object might be viewed as having more than one strength of emergence; the strength of emergence being related to the way in which we model the object, or to the complexity of the components we use within the model. Traditionally, modeling was oriented towards the description of an object in such a way that the property of interest would be viewed as weakly emergent as possible: the less the component properties are state dependent, the easier it should be to deduce them from the knowledge of element properties in isolation, the less we need to know about the system as a whole, and the easier it should be to parameterize the model. The model would then also be more universal, less state-dependent and, consequently, more robust against the changes of initial and boundary conditions.

However, we believe that this reductionist approach is flawed from the perspective of the fundamental aim of Biology, i.e., understanding life. Vis-à-vis this aim, it would be more prudent to start with constructing a model with a high number of components and with a high degree of state-dependency of each component property; a model that is realistic rather than simple (Westerhoff et al., 2009b). Importantly, the emergence of properties then becomes dependent upon the network itself and not on the assumptions used to drive a simulation towards a predicted outcome. Although the construction of such a complex model might be difficult, the payoff is a much improved probability of discovering actual system properties in the real-life system and of grasping new design principles (Teusink et al., 1998; Bakker et al., 2000; Hornberg et al., 2005; Haanstra et al., 2008; Kolodkin et al., 2010; Boogerd et al., 2011). *In silico* experimentation with computer model have proved of immense value here. They have helped discover important functions of complexity such as the summation law of metabolic control (Kacser and Burns, 1973), the connectivity law for concentration control (Westerhoff and Chen, 1984), the function of trehalose inhibition of hexokinase in yeast (Teusink et al., 1998), the function of the glycosomal membrane in *Trypanosoma brucei* (Bakker et al., 2000; Haanstra et al., 2008), and the function of NR in the cytosol (Kolodkin et al., 2010). In addition, the added levels of complexity (and hence of biological realism) may help identify unexpected targets for intervening in the biological reality, thus driving drug discovery forward in a network-targeting, rather than a molecule-targeting, paradigm (Bakker et al., 1999).

In fact, when building systems biology models, we should be led by the principle of the “biological binocular,” rather than by the law of parsimony (Kolodkin and Westerhoff, 2011). Let us discuss this issue in more detail. The law of parsimony suggests that “the simplest explanation is most likely the correct one.” Consider a phenomenon which can be explained in two different ways, the first explanation requiring entities (terms, factors, transformations etc.) A, B and C, and the second explanation requiring entities A, B, C and D. Assume that both explanations give the same result. Then, according to the law of parsimony, entity D is unnecessary and the first (the simpler) explanation is most likely the correct one. The law of parsimony is based on “Occam's razor” – a heuristic method consisting of “shaving away” unnecessary assumptions. William of Occam (1285–1349) formulated his principle as follows: “One should not postulate (pose) more things without necessity” (*Pluralitas non est ponenda sine necessitate*). After Occam's death, this statement was modified to “Entities should not be increased (multiplied) without necessity” (*Entia non sunt multiplicanda praeter necessitatem*) (Murta, 2012). The application of Occam's razor provided then (early fourteenth century) much-needed motivation to view the behavior of physical objects as being determined by simple physical laws rather than by divine intervention. And this continues to be an essential precondition for modern science. However, when calling a generalization a law, one implies that the generalization may be validated, e.g., it may be empirically observable, and should always be true, at least in its defined, somewhat extensive regime of validity. The absence of repeatable contradictory observations does not guarantee that this generalization is a law rather than a particular consequence from other, more universal laws. The law of gravity constitutes an example. The universality of the force of gravity is presently being questioned. It is now suggested that gravity could be described as a phenomenon emerging from more fundamental forces, such as the entropic force (Verlinde, 2011).

The principle of Occam's razor has never been proven to be universally valid. It will never be proven either that the simpler explanation is always the right one: it has been shown repeatedly that the more complex explanation can be true; we only need refer to the examples of chemiosmotic coupling (Mitchell, 1961), the control of metabolic fluxes (Groen et al., 1982), and general relativity (Einstein, 1961). There are examples in physics where the simplest explanation turned out to be the more realistic one. However, this does not imply that those explanations were more realistic because of any universal tendency towards simplicity. On the contrary, the second law of thermodynamics suggests that there is a tendency to complexity rather than to simplicity (Westerhoff et al., 2009b). An important issue here is that whereas physics may study the unlimited, biology studies large but limited systems. Instead of shaving *pluralitatem* away, one should be interested in discovering, in seeing, in distinguishing these *pluralitates* and in taking them into account: “One should not remove things without necessity” (*Pluralitas non est eliminanda sine necessitate*) (Kolodkin and Westerhoff, 2011). Then the law of parsimony will become also the law of completeness: “If entities A, B, C and D (e.g., proteins) are discovered in a system S (e.g., cell, organism, ecosystem) for the fitness of which A, B, C, and D are all known to be essential, and if some properties of system S can be equally well explained either via A, B and C or via A, B, C and D, then the more complex explanation is most likely the correct one”; in the language of the concept of emergence, we should assume emergence to be as strong as possible.

## Concluding remarks

Modeling, in its general sense, is an everyday process that our brains employ to make sense of the physical world around us. When applied to science, and specifically in the study of biological networks, modeling may take the form of describing these biological processes with mathematical models that help us unravel the intricacies and complexities of the convoluted and highly interacting biological networks we have to deal with. The application of modeling in studying complex biological networks does not need to rob us of our ability to infer design principles and observe the emergent properties of the system. By reconstructing the emergence of the properties of a system, we can identify its design and understand the mechanisms underlying its function. *In silico* experimentation in support of conceptual analyses can help here.

The example of neurodegenerative diseases is well suited for the application of the systems biology approach. Such diseases are complicated, multifactorial constructs, and improving our knowledge of their function requires understanding of the emergent properties of the interacting components of the system that is the human body. Since disease (a pathological shift of homeostasis) emerges from the perturbation of the network as a whole, the biomolecular network of every individual is unique and this is observed when similar disease-producing agents cause different individual pathologies. Consequently, a personalized model for every patient may be required for therapies to become universally effective (Lehrach et al., 2011). This fits well with the P4 medicine concept of contemporary medicine (Tian et al., 2012).

The “silicon human,” as well as the *in silico* reconstruction of biological emergence may prove invaluable for the comprehensive understanding of body functioning, for novel paradigms of drug discovery, and for the development of patient-specific treatments, especially in the case of multifactorial network diseases such as neurodegenerative diseases, including Parkinson's disease.

### Conflict of interest statement

The authors declare that the research was conducted in the absence of any commercial or financial relationships that could be construed as a potential conflict of interest.
